# Supplementary material for: Maintenance of service delivery during medical countermeasures deployment: The association between the COVID-19 vaccine rollout and continuity of routine childhood immunization services in Uganda
Source: PLOS Glob Public Health. 2025 Jun 13;5(6):e0004731. doi: 10.1371/journal.pgph.0004731 (PMC12165430; doi:10.1371/journal.pgph.0004731)
Supplement: S1 File — (DOCX) [file pgph.0004731.s001.docx]

**S1 File: Details of Key Informants Interviewed:**

| **#** | **Level of Health Facility** | **Gender** | **Age Category** | **Education level** | **Occupation** | **Position** |
| --- | --- | --- | --- | --- | --- | --- |
|  | Public HC IV | Female | 30-35 | Diploma in Nursing | Registered Nurse | EPI focal person |
|  | Public HC IV | Female | 25-29 | Registered Midwife | Midwife | Midwife |
|  | Public HC IV | Female | 50-55 | Certificate in Nursing | Nurse | EPI focal person |
|  | Public HC III | Female | 30-35 | Certificate in Nursing | Nurse | EPI focal person |
|  | Public HC III | Female | 40-45 | Diploma in clinical Medicine | Clinician | Acting in-charge for the public health department |
|  | Public HC IV | Female | 35-40 | Degree in Midwifery | Midwife | EPI focal person |
|  | Public HC IV | Female | 25-30 | Diploma in Midwifery | Midwife | Elimination of Mother to Child Transmission Focal Person |
|  | Public HC III | Female | 25-30 | Diploma in Medical Lab | Nurse | EPI focal person |
|  | Hospital Private-for-profit | Female | 30-35 | Certificate midwifery | Enrolled Midwife | EPI midwife |
|  | Public HC IV | Female | 30-35 | Diploma in psychiatric nursing | Nurse | In-charge EPI |
|  | Public HC IV | Female | 25-30 | Diploma in midwifery | Registered Midwife | Midwife |
|  | Public HC III | Male | 35-40 | Clinical officer | Clinician | Assistant EPI focal person |
|  | Public HC III | Female | 40-45 | Certificate in Nursing | Nurse | Immunisation Nurse |
|  | Public HC III | Female | 45-50 | Certificate in Nursing | Nurse | Nurse |
|  | Public HC III | Female | 35-40 | Certificate in Nursing | Nurse | Nurse |
|  | HC IV Private-for-profit | Female | 25-30 | Diploma in Midwifery | Midwife | Midwife |
|  | Public HC IV | Female | 25-30 | - | Technician | Cold chain Technician |
|  | Public HC III | Male | 55-60 | Certificate in Nursing | Nurse | Nurse |
|  | Public HC III | Female | 20-25 | Certificate in Nursing | Nurse | Assistant EPI focal person |
