# Supplementary material for: Maintenance of service delivery during medical countermeasures deployment: The association between the COVID-19 vaccine rollout and continuity of routine childhood immunization services in Uganda
Source: PLOS Glob Public Health. 2025 Jun 13;5(6):e0004731. doi: 10.1371/journal.pgph.0004731 (PMC12165430; doi:10.1371/journal.pgph.0004731)
Supplement: S2 File — (DOCX) [file pgph.0004731.s002.docx]

Segmented Poisson regression outputs from STATA.

|  | **Trends [95% CI] Jan 2018-Feb 2020** | **Trends [95% CI] Mar 2020-Feb 2021** | **Trends [95% CI] Mar 2021-Dec 2022** | **Trends [95% CI] Mar 2020-Dec 2022** |
| --- | --- | --- | --- | --- |
| DPT3 | -0.0002449 [-0.0032719-0.0027821] | 0.0164935 [0.0019675-0.0310194] | 0.0073329 [0.0025875-0.0120784] | 0.0080031 [0.0050084-0.0109978] |
|  | 0.999 [0.992-1.006] | **1.039 [1.005-1.074]** | **1.017 [1.006-1.028]** | **1.019 [1.012-1.026]** |
| BCG | 0.0035433 [-0.0009277-0.0080142] | 0.0041804 [-0.0102092-0.0185699] | 0.0052675 [-0.0005235-0.0110585] | 0.0076356 [0.0051561-0.0101152] |
|  | 1.008 [0.998-1.019] | 1.010 [0.977-1.044 | 1.012 [0.999-1.026] | **1.018 [1.012-1.024]** |
| Polio 0 | 0.0085109 [0.0043016-0.0127202] | 0.008428 [-0.0049619-0.0218178] | 0.0083717 [0.0040945-0.012649] | 0.0105739 [0.0083594-0.0127883] |
|  | **1.020 [1.010-1.030]** | 1.020 [0.989-1.052] | **1.019 [1.009-1.030]** | **1.025 [1.019-1.030]** |
| Polio 1 | 0.0023374 [-0.0026443-0.0073191] | 0.0111474 [0.0000467-0.0222482] | 0.009945 [0.0066424-0.0132467] | 0.0096897 [0.0075933-0.0117862] |
|  | 1.005 [0.994-1.017] | **1.026 [1.000-1.053]** | **1.023 [1.015-1.031]** | **1.023 [1.018-1.028]** |
| Polio 2 | 0.004381 [0.0004411-0.0083209] | 0.0178872 [0.0038269-0.0319475] | 0.0091913 [0.0054734-0.0129093] | 0.0092452 [0.0064134-0.012077] |
|  | **1.010 [1.001-1.019]** | **1.042 [1.009-1.076]** | **1.021 [1.013-1.030]** | **1.022 [1.015-1.028]** |
| Polio 3 | 0.003476 [-0.0001669-0.0071189] | 0.0157634 [0.0005766-0.0309502] | 0.0083975 [0.0033204-0.0134746] | 0.0086445 [0.00569-0.011599] |
|  | 1.008 [0.999-1.017] | **1.037 [1.001-1.074]** | **1.019 [1.008-1.032]** | **1.020 [1.013-1.027]** |

Interrupted Times Series analysis Results from R

|  | **Intercept** | **Time** | **Covid** | **Post Covid** | **Vaccine** | **Post Vaccine** |
| --- | --- | --- | --- | --- | --- | --- |
| DPT3 | 8395.730 [8309.115293 8482.344016] | -3.812 [-9.842247 2.219093] | -1667.110 [-1964.152528 -1370.067983] | 208.814 [175.424264 242.204673] | -1172.118 [-1484.417374 -859.819373] | -120.850 [-151.945912 -89.754356] |
| BCG | 6990.778 [6895.948981 7085.60672] | 17.809 [11.165259 24.45270] | -809.865 [-1136.566954 -483.16343] | 166.68764 [-15.421126 58.38246] | 331.492 [-8.283771 671.26734] | 4.326 [-30.362149 39.01352] |
| Polio 0 | 5015.378 [4944.719486 5086.037005] | 48.753 [43.817217 53.688372] | -1193.273 [-1439.919395 -946.625925] | 25.605 [-2.165949 53.375460] | 140.082 [-119.864384 400.028677] | -18.850 [-44.640432 6.940333] |
| Polio 1 | 7627.194 [7455.58780 7798.80098] | 40.201 [28.14188 52.25969] | -1736.207 [-2220.63081 -1251.78358] | 121.244[66.05405 176.43319] | -787.132 [-1266.09754 -308.16550] | -67.874 [-122.79962 -12.94829] |
| Polio 2 | 7627.194 [7455.58780 7798.80098] | 40.201 [28.14188 52.25969] | -1736.207 [-2220.63081 -1251.78358] | 121.244 [66.05405 176.43319] | -787.132 [-1266.09754 -308.16550] | -67.874[-122.79962 -12.94829] |
| Polio 3 | 7286.522 [7179.48943 7393.55524] | 37.175 [29.67109 44.67944] | -1875.091 [-2245.50845 -1504.67390] | 188.476 [146.62060 230.33056] | -1336.025 [-1721.53924 -950.51014] | -130.803 [-170.11369 -91.49230] |
