# Supplementary material for: Maintenance of service delivery during medical countermeasures deployment: The association between the COVID-19 vaccine rollout and continuity of routine childhood immunization services in Uganda
Source: PLOS Glob Public Health. 2025 Jun 13;5(6):e0004731. doi: 10.1371/journal.pgph.0004731 (PMC12165430; doi:10.1371/journal.pgph.0004731)
Supplement: Attachment — Submitted filename: Response to Reviewers Comments_1.docx [file pgph.0004731.s006.docx]

29 April 2025

PGPH-D-25-00240
Maintenance of Service Delivery During Medical Countermeasures Deployment: The Effect of the COVID-19 Vaccine Roll Out on Routine Childhood Immunization Services in Uganda
PLOS Global Public Health

Dear Dr. Edina Amponsah-Dacosta

Thank you for reviewing our manuscript, “Maintenance of Service Delivery During Medical Countermeasures Deployment: The Effect of the COVID-19 Vaccine Roll Out on Routine Childhood Immunization Services in Uganda,” and giving us the opportunity to improve it. Below are point by point responses to each of the comments raised by the editor and both reviewers:

**Comments from the Editor**

**Comment:** The authors should ensure that the methodological limitations of the study are fully addressed, preferably in a dedicated paragraph as part of the Discussion section. This should include consideration for any biases introduced and how these were managed. Any impact on interpretation / generalizability of findings should be underscored.

**Response: This is a pertinent comment from the editor and we have included the methodological limitations in the last paragraph of the discussion.**

**Comment:** Please include the following items when submitting your revised manuscript:

- A rebuttal letter that responds to each point raised by the editor and reviewer(s). You should upload this letter as a separate file labeled 'Response to Reviewers'.
- A marked-up copy of your manuscript that highlights changes made to the original version. You should upload this as a separate file labeled 'Revised Manuscript with Track Changes'.
- An unmarked version of your revised paper without tracked changes. You should upload this as a separate file labeled 'Manuscript'.

**Response: A rebuttal letter, marked-up copy of the manuscript and an unmarked copy have all been provided as guided by the editor.**

**Comment:** Please include a complete copy of PLOS’ questionnaire on inclusivity in global research in your revised manuscript. Our policy for research in this area aims to improve transparency in the reporting of research performed outside of researchers’ own country or community. The policy applies to researchers who have travelled to a different country to conduct research, research with Indigenous populations or their lands, and research on cultural artefacts. The questionnaire can also be requested at the journal’s discretion for any other submissions, even if these conditions are not met.  Please find more information on the policy and a link to download a blank copy of the questionnaire here: https://journals.plos.org/globalpublichealth/s/best-practices-in-research-reporting. Please upload a completed version of your questionnaire as Supporting Information when you resubmit your manuscript.

**Response: We thank the Editor for this comment and have now included the PLOS questionnaire on inclusivity in global research as part of the supporting information. We have also included a sub-section “Inclusivity in Global Research” under ethical considerations and referred to this specific supplementary information.**


**Response to Comments from Reviewer #1**

**Comment:** The paper addresses a critical, yet often overlooked, aspect of pandemic response — how the introduction of emergency countermeasures like COVID-19 vaccines can inadvertently disrupt essential routine health services. This study fills an important gap in the literature and is especially relevant for health systems in low- and middle-income countries navigating limited infrastructure, human resources, and competing health priorities.

**Response: We thank the reviewer for taking the time to read through our work and providing invaluable inputs that will definitely improve the quality of the paper.**

**Comment:** What Works Well: The use of a sequential explanatory mixed-methods design is appropriate and well-justified. The quantitative data establish clear trends, while the qualitative findings give much-needed depth and context, capturing the lived experiences of health workers and caregivers during the pandemic.

**Response: We thank the reviewer for taking the time to read through our work and providing invaluable inputs that will definitely improve the quality of the paper.**

**Comment:** I also commend the authors for grounding their analysis in established frameworks (WHO Health System Building Blocks, Health Systems Dynamics Framework), which adds structure and relevance to the findings.

**Response: We thank the reviewer for taking the time to read through our work and providing invaluable inputs that will definitely improve the quality of the paper.**

**Comment:** The manuscript is well-written, coherent, and the policy implications are clearly drawn. The topic is timely, the methods are solid, and the findings have both practical and scholarly value.

**Response: We thank the reviewer for taking the time to read through our work and providing invaluable inputs that will definitely improve the quality of the paper.**

**Comment:** Suggestions for Improvement: Although the study is strong, a few revisions could further improve its clarity, precision, and impact.

1. Clarify Language Around Causality. The quantitative analysis — particularly the interrupted time series — is appropriate for exploring associations over time. However, the manuscript occasionally leans into causal language (“impact,” “effect”) which may overstate the findings. I recommend using more cautious terms like “associated with” or “linked to” unless additional modeling or control for confounders is introduced.

**Response: We agree with this insightful suggestion from the reviewer and have removed any language that insinuates impact or effect and replaced with “associated with.”**

**Comment:** 2. Reconsider Emphasis on One-Month Comparison. The use of percentage changes between March and April 2021 to demonstrate changes in vaccine uptake is informative but limited. One month is a narrow window, and seasonal or short-term factors could influence results. The ITS graphs provide a more complete picture and should be emphasized more strongly in both the results and discussion. If possible, consider expanding the quantitative interpretation to reflect longer-term trends.

**Response: We have added more detail to the interpretation of the graphs from the ITS analysis to indicate that beyond the immediate drops in service use associated with the vaccine rollout, it appears that there is steady recovery across the indicators. We have also further discussed this in the discussion section that the steady recovery might be as a result of the interventions that were implemented to sustain both the demand and delivery of services.**

**Comment:** 3. Improve Integration Between Quantitative and Qualitative Results. Currently, the quantitative and qualitative findings are somewhat compartmentalized. The discussion would benefit from more explicit connections between the two — for example, linking the observed declines in immunization to specific barriers like cold chain competition, workload pressures, or community hesitancy described by participants.

**Response: We agree with the reviewer and have integrated the quantitative with the qualitative by stating how the reductions in service use observed in the quantitative analysis could be explained by the challenges to delivery and use in the qualitative findings, and that the interventions implemented to sustain demand and delivery could explain the sustained recovery across the indicators analyzed. We thank the reviewer for this suggestion.**

**Comment:** 4. Visual Clarity of Figures.The ITS graphs are helpful but could be improved. Enhancing the resolution, labeling axes more clearly, and adding vertical reference lines (e.g., to mark the vaccine rollout or pandemic start) would make the data more reader-friendly.

**Response: We thank the reviewers for this comment and have now provided more clear graphs for the interrupted times series analysis where we have increased the thickness of the vertical lines indicating the vaccine rollout and start of the pandemic. We have also added the graphs as images to preserve the resolution as opposed to merging them which lowers the quality of the images.**

**Comment:** 5. Data Sharing and Transparency. The Data Availability Statement suggests that data are included as supplementary material. However, it would be good to confirm that the raw data used in the ITS analyses (i.e., monthly vaccine counts) are available in a downloadable format (e.g., Excel or CSV). If sharing full qualitative transcripts isn’t possible for ethical reasons, a brief justification would be helpful.

**Response: We agree with the reviewer and have provided the aggregate data used for the quantitative analysis as well as the codes and themes that emerged from the qualitative analysis within the Excel file.**

**Comment:** Minor Notes for improvement: 1. Consistency in Terminology and Style: The manuscript alternates between phrases like “COVID-19 vaccine rollout,” “COVID-19 vaccination,” and “deployment of COVID-19 vaccines.” While all of these are technically correct, consistent use of a single term (e.g., “COVID-19 vaccine rollout”) would improve readability and flow. Suggested action: Decide on one preferred phrase and apply it consistently throughout the text, including the abstract, results, and discussion sections.
Example: Line 29: “…the COVID-19 vaccination rollout…” Line 388: “…the subsequent rollout of COVID-19 vaccines…” These could both be rephrased to maintain consistency (e.g., use "COVID-19 vaccine rollout" throughout).

**Response: We thank the reviewer for this comment and have now used one term “COVID-19 vaccine rollout” as suggested by the reviewer in the entire paper.**

**Comment:** 2. Reference Formatting and URL Display: Several references (particularly government or WHO documents) contain long URLs that are broken across multiple lines or presented in a cluttered format, which can disrupt the reading experience and reduce professionalism. Suggested action: Use hyperlink text (e.g., "Uganda Statistical Abstract 2021") instead of pasting long URLs directly. Also, ensure uniform citation formatting (e.g., consistent punctuation, date format, journal names). Example: Ref 14: “https://www.ubos.org/wp- content/uploads/publications/01_20222021_Statistical_Abstract.pdf” → could be replaced with: Uganda Bureau of Statistics. (2021). Statistical Abstract. <https://www.ubos.org/wp-content/uploads/publications/01_20222021_Statistical_Abstract.pdf>

**Response: We thank the reviewer for this comment. The references have been edited and improved as guided.**

**Comment:** Also ensure references to journal articles include full volume, issue, and page numbers consistently across the list.

**Response: We thank the reviewer for this comment. We have reviewed the reference list for consistency and adherence to journal guidelines.**

**Comment:** 3. COREQ Checklist – Transparency on “Not Applicable” Items: The authors have done well to complete the COREQ checklist. However, several items are marked "Not Applicable" (e.g., transcript return, participant checking), and while this may be appropriate, it's important to briefly justify these omissions in the manuscript itself — ideally in the methods section or a brief note in the limitations. This is especially relevant for qualitative research, where transparency and reflexivity are central to trustworthiness. Suggestions on How to Address This: For transcript return: “Transcripts were not returned to participants for comment or correction due to logistical constraints and time limitations. However, efforts were made to ensure accuracy through verbatim transcription and note-taking during interviews.”
**Response: We thank the reviewer for this comment and the time taken to provide the suggestion. We have made the change as suggested by the reviewer.**

**Comment:** For participant checking of findings: “Findings were not formally validated with participants after analysis; however, triangulation across multiple data sources and use of a structured coding framework supported the credibility of results.” These clarifications would reassure readers and reviewers that the researchers were mindful of qualitative rigor, even if certain validation steps were not feasible.

**Response: We thank the reviewer for this comment and the time taken to provide the suggestion. We have made the change as suggested by the reviewer.**

**Comment:** Although these are relatively small issues, addressing them would improve the readability, professionalism, and transparency of the manuscript. They do not detract from the strength of the study but will contribute to its polish and clarity for publication.

**Response: We thank you so much for the time you have take to not only review the paper but also give specific recommendations of improving the quality of the paper. We appreciate this very much.**

**Response to Comments from Reviewer #2**

**Comment:** Interesting study and an important contribution to the literature on how COVID-19 impacted routine immunisation. Overall, I think the language needs to be made a bit clearer. i.e. outreaches should just be outreach and there are some sentences that are difficult to understand.
**Response: We thank the reviewer for this comment. We have reviewed the entire paper and removed unclear language. This comment was also raised by reviewer #1 about the consistency in terminology to make the paper more understandable. For example we have removed terms like “the rollout of COVID-19 vaccines”,” introduction of COVID-19 vaccines” and “COVID-19 vaccination rollout” and replaced these with one term “COVID-19 vaccine rollout” in the entire paper. We have also replaced outreaches with outreach as suggested by the reviewer.**

**Comment:** Line 110 – underlying 'health' conditions?
**Response: We have edited underlying to clarify “underlying health conditions” as suggested by the reviewer.**

**Comment:**Line 122-123 - this doesn't add anything to know that they have been described in pervious papers. I would just stick with the example you have listed and delete this sentence.
**Response: We thank the reviewer for this comment. The rationale for pointing readers to where the system has been described is because there are additional details such as the frequency of data collection, personnel involved in data collection, when the system was rolled out and the coverage of the system all of which are not described in the current paper. The purpose of including the citations is for a reader to access this detail without necessarily repeating it in the current manuscript.**

**Comment:** In your methods - you need to include whether the facilities were government owned, not-for-profit etc. as you discuss this in your results but I couldn't link it back to your methods.

**Response: This is a good comment from the reviewer and had been an oversight on our part. The details of whether private or public/ government facility have been included in the description of every facility for each key informant. These details are part of Supplementary Material 1 that describes each key informant.**

**Comment:** WHO building blocks - you say you used this to guide the surveys and interviews but then in your analysis you have not used it to frame your results. Where do all the challenges and strategies you have identified fit into the WHO building blocks framework?

**Response: This is a great comment from the reviewer. The reason the results are not structured under the WHO building blocks framework is because we employed an abductive approach to analysis. This allows for identification of themes deductively using a framework but with the flexibility to identify themes and patterns beyond this framework. For example, issues related to health workforce and the challenges related to cold chain infrastructure are in perfect alignment with the WHO health system building blocks. However, issues like perceptions about routine vaccines due to the COVID-19 vaccine rollout would’ve been missed. A purely deductive approach would have limited the depth of the qualitative analysis. An abductive approach is a balance between deductive analysis using a framework and inductive analysis to identify themes that are pertinent to a research question. In the data analysis section, we provided a rationale for the use of abductive data analysis.**
